# A novel mutation of the *PAX3* gene in a Chinese family with Waardenburg syndrome type I

**DOI:** 10.1002/mgg3.798

**Published:** 2019-06-12

**Authors:** Jing Ma, Ken Lin, Hong‐chao Jiang, Yanli Yang, Yu Zhang, Guilian Yang, Hao Sun, Cheng Ming, Xianyun Bi, Tiesong Zhang, Biao Ruan

**Affiliations:** ^1^ Department of Otolaryngology, Head & Neck Surgery, Kunming Children’s Hospital Kunming China; ^2^ Yunnan Pediatric Institute, Kunming Children’s Hospital Kunming China; ^3^ Department of Otolaryngology First Hospital of Kunming Medical University Kunming China; ^4^ Yunnan Rehabilitation School For Children With Hearing Impairment Kunming China; ^5^ Institute of Medical Biology, Chinese Academy of Medical Sciences & Peking Union of Medical College Kunming China

**Keywords:** gene mutation, hereditary deafness, *PAX3*, Waardenburg syndrome type I

## Abstract

**Background:**

To analyze the clinical phenotypes and genetic variants of a Chinese family with Waardenburg syndrome (WS) and to explore the possible molecular pathogenesis of WS.

**Methods:**

The clinical data from a patient and his family were collected. The genomic DNA of the patient and his family was purified from their peripheral blood. All exons and flanking sequences of the *MITF, PAX3, SOX10, SNAI2, END3,* and *EDNRB* genes were investigated through high‐throughput sequencing. Based on the results of high‐throughput sequencing, genetic variants in the patient and his family were verified and analyzed by Sanger sequencing.

**Results:**

The patient was diagnosed with typical WS1 that manifested in hearing impairment, inner canthus ectopia and heterochromic iris. Sanger sequencing revealed the pathogenic heterozygous c.420‐424de1CGCGGinsTTAC mutation in the *PAX3* gene in the proband, which is a frameshift mutation that changed the amino acid sequence of the *PAX3* protein from AVCDRNTVPSV to YSVIETPCRQ* (* refers to a stop codon) from amino acids 141–151. The stop codon induced by this mutation resulted in the truncation of the *PAX3* protein. The same mutation sites were also found in the mother and younger sister of the proband. No previous report of this mutation was found in the Human Gene Mutation Database.

**Conclusion:**

The novel heterozygous c.420‐424de1CGCGGinsTTAC mutation is the molecular pathological cause for WS1 in our patient. The clinical and genetic characterization of this family with WS1 elucidated the genetic heterogeneity of *PAX3* in WS1. Moreover, the mutation detected in this case has expanded the database of *PAX3* mutations.

## 1 INTRODUCTION

Waardenburg syndrome (WS), also known as auditory‐pigmentary syndrome, is the most common cause of syndromic deafness. It is a congenital genetic disease mainly caused by monogenetic variants that is mostly inherited through autosomal dominance with incomplete penetrance. The incidence of WS is 1/42,000, which contributes to 2%–5% of congenital deafness. The morbidity of WS in deaf individuals is 0.9%–2.8% (Nayak & Isaacson, 2003; Peker, Ergil, & Ozturk, 2015). WS patients manifest dysfunction in multiple areas, including the hair, skin, eyes, ears, craniofacial region, gastrointestinal tract, urogenital tract, and central nervous system. Typical characteristic phenotypes of WS include sensorineural deafness, skin pigmentation disorders, frontal or early leukotrichia, and heterochromic iris. WS is classified into four types (WS1, WS2, WS3, and WS4) according to their different clinical manifestations (Bondurand et al., 2007; Pingault et al., 2010). WS1 (OMIM #193500) and WS2 (OMIM #193510) are more common than WS3 and WS4 and cause preverbal sensorineural deafness and heterochromic iris. WS1 is often accompanied by ectopia of the inner canthus (W index > 1.95), while WS2 is not. Patients with WS3 (Klein‐Waardenburg syndrome, OMIM #148820) exhibit all the symptoms of WS1 in addition to upper limb deformity. Similarly, patients with WS4 (Waardenburg‐Hirschsprung disease, OMIM #277580) exhibit all the symptoms of WS2 in addition to megacolon or gastrointestinal atresia. WS has a high degree of genetic heterogeneity, and different patients in the same family can exhibit different clinical manifestations due to variations in the penetrance of causative genes. Six genes are related to WS, including *PAX3* (paired box gene 3, OMIM #606597), *MITF* (microphthalmia‐associated transcription factor, OMIM #156845), *SNAI2* (snail homolog 2, OMIM #602150), *EDN3* (endothelin‐3, OMIM #131242), *EDNRB* (endothelin receptor type B, OMIM #131244), and *SOX10* [SRY (sex determining region Y)‐box 10, OMIM #602229], which are located on chromosomes 2, 3, 8, 13, 20, and 22, respectively. Interactions between these related genes play important roles in WS pathogenesis. *PAX3* is a crucial pathogenic gene of WS1 and WS3 (Tassabehji et al., 1993), while WS2 and WS4 are caused by mutations in the *SNAI2, MITF, SOX10, EDNRB,* and *EDN3* genes. Among them, the mutation of *SOX10* is the main pathogenic factor in Chinese patients with WS2 (Chen et al., 2010; Ma et al., 2016; Zheng et al., 2018).

Due to the diversity of WS manifestations and the molecular genetics mentioned above, exploring the clinical characteristics of a family with WS and detecting the causative genes and their variants can facilitate the development of methods to genetically diagnose WS and the further study of WS pathogenic mechanisms. In this report, we analyzed the relative genetic variants in a Chinese family with WS1 and explored the possible molecular pathogenesis of WS1.

## 2 MATERIALS AND METHODS

### 2.1 Case data

The proband was from Yunnan, China, suffered from congenital profound sensorineural deafness and received a cochlear implant in Kunming Children's Hospital. Clinical data from the principle members in the proband's family were collected through questionnaires. All genetic diagnoses and prenatal genetic diagnoses were performed with obtained consent from the patient and his parents. Additionally, 50 healthy individuals, including 30 males and 20 females aged 12–60, were enrolled as healthy controls, and their genomic DNA was collected from the DNA library of the Molecular Diagnosis Center of Deafness, Yunnan Institute of Pediatric Research. Otology and physical examinations were also performed on all individuals in the control group. This study was approved by the Medical Ethics Committee of Kunming Children's Hospital.

### 2.2 Assessment and diagnosis of clinical manifestations

The patient was completely examined in the areas of intelligence, audiology, ophthalmology, hair, skin, limb joints, and digestive system. The distances between the inner canthus, outer canthus, and pupils were calculated and quantified as the W index as follows: where A = inner canthic diameter, B = pupillary distance, and C = outer canthic diameter. Otoacoustic emission, acoustic immittance, auditory brain stem response (ABR), multiple steady‐state responses (ASSR), and verbal hearing aid tests were performed to assess the audition of the patient. Additionally, a preoperative hearing speech level assessment, temporal CT, and cranial MRI were also performed.

### 2.3 DNA library preparation

Genomic DNA was extracted from the peripheral blood using the DNA Extraction kit (QIAamp DNA Blood Midi Kit, Qiagen, Shanghai, China) following the manufacturer's instructions. Qualified genomic DNA was fragmented randomly, and fragments with lengths from 150–250 bp were purified. T4 DNA polymerase, T4 phosphorylated polynucleotide kinase, and E. coli DNA polymerase Klenow fragments were used to repair the ends of the DNA fragments. Then, “A‐tails” were ligated to the DNA fragments following the instructions for a sequenator from Illumina. Adaptors from Illumina were ligated to the DNA fragments, which were subsequently purified by magnetic beads.

### 2.4 Target gene capture

Adapted DNA templates were amplified through PCR before being captured. PCR products were hybridized with a designed GenCap Custom Enrichment Kit (MyGenostics, Beijing, China) under appropriate conditions for 22 hr. Then, target DNA regions were captured by probes, and nonhybridized regions were washed off. The quantity of the probe‐captured target DNA fragments was significantly amplified through another round of capture PCR.

### 2.5 Bioinformatics analysis

The chip‐captured fields in this experiment included all exons and their flanking sequences (approximately 100 bp) in the human genome. Raw data from high‐throughput sequencing were produced through Illumina Pipeline (version 1.8.2). Low‐quality data were removed, and “clean” reads were aligned with the reference sequence of the human genome (UCSC, hg19) through BWA (Burrows‐Wheeler aligner). SNPs and INDELs were collected by SOAPsnp and GATK software, respectively. By reference to the dbSNP database, the 1000 genomes database, the ESP database (NHLBI Exome Sequencing Project) and data from 50 healthy control individuals, mutation sites with frequencies less than 0.05 were selected as suspicious pathologic mutations.

### 2.6 Sanger sequencing

Genomic DNA from the parents of the proband was extracted from venous blood for Sanger sequencing. Based on the results of high‐throughput sequencing, primers for suspicious pathologic mutations were designed using the online software PRIMER3 (forward primer: 5′‐CCTCCCTCCATAAAGTGCCA‐3′, reverse primer: 5′‐CTGCCCGCCTGTTCTCTTAA‐3′). PCR amplification was performed with the following procedure: an initial denaturation at 95°C for 5 min; 34 cycles of denaturation at 94°C for 30 s, renaturation at 60°C for 30 s, and extension at 72°C for 45 s; and a final extension at 72°C for 5 min. The PCR products, which were 420 bp in length, were analyzed through gel electrophoresis and then purified. Capillary electrophoretic sequencing was performed through the ABI PRISM 3730 sequenator, and mutations were analyzed. The Sanger sequencing results were aligned to the *PAX3* reference sequence (NM_181457.3) with SeqMan7.1 software.

### 2.7 Validation of mutations

The detected mutations were analyzed in the NCBI dbSNP database, the HapMap database, and the 1000 genomes database, and known polymorphic sites were excluded. Then, the mutations were searched for in the Human Gene Mutation Database (HGMD, http://www.hgmd.org/) and the PubMed database (http://www.ncbi.nlm.nih.gov/pubmed/) to determine whether they had been reported. The functions of proteins with novel mutations were predicted with Mutation Taster software.

## 3 RESULTS AND ANALYSIS

### 3.1 Clinical manifestation analysis of the proband and his family

**Figure 1:**
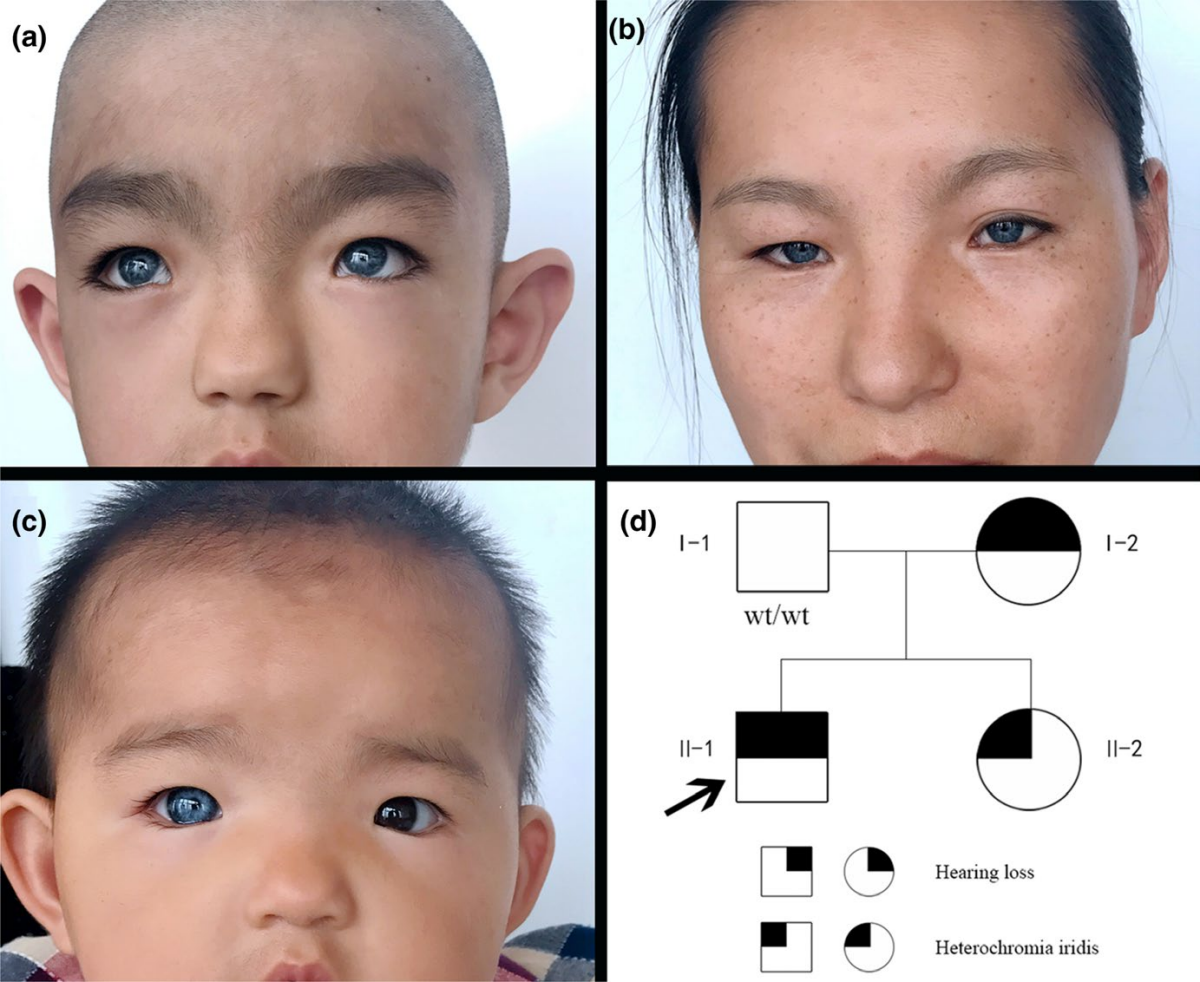
Clinical features and Family pedigree of the proband. Clinical features of the proband included iris heterochromia in both eyes (a); Clinical features of the proband's mother included iris heterochromia in both eyes (b); Clinical features of the proband's younger sister included iris heterochromia in the right eye (c); Family pedigree of the proband (d)

**Figure 2:**
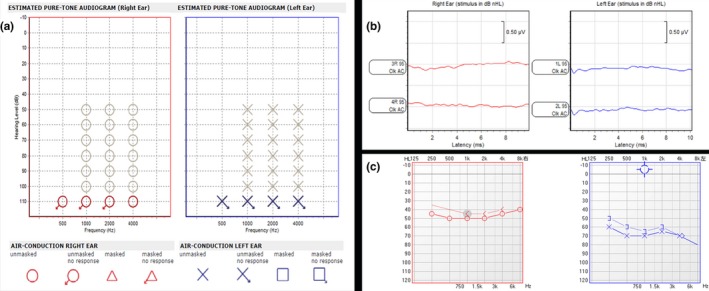
Clinical audiological examination. ASSR confirmed profound bilateral congenital sensorineural hearing loss (a); ABR confirmed profound bilateral congenital sensorineural hearing loss (b); Air conduction hearing threshold of the mother. Right ear, 50 dB HL; left ear, 70 dB HL. dB HL, decibel hearing level (c). ABR, auditory brain stem response; ASSR, auditory steady‐state responses

The proband was 5 years and 4 months old and suffered from congenital sensorineural deafness and profound bilateral full‐frequency hearing loss. He was normally delivered full‐term with a bilateral blue iris, broadened inner canthi, a nasal bridge pit, and a wide nasion at birth (Figure 1a). Clinical audiological examination showed failed bilateral otoacoustic emissions, all bilateral ABR thresholds over 95 dB nHL (Figure 2b), and ASSR 500–4,000 Hz thresholds over 110 dB HL (Figure 2a). Temporal CT and cranial MRI showed no obvious abnormalities. The measured inner canthic diameter was 40 mm, the outer canthic diameter was 94 mm, the pupillary distance was 60 mm, the W index was 2.2834 (over 1.95), and the patient was clinically diagnosed with WS1. No dysfunction was detected in the vision and intelligence of the proband, no abnormality was detected in the hair and limb joints of the proband, and no history of toxic medications was known. The mother of the proband was 40 years old and exhibited a bilateral blue iris, a face scattered with freckles and frontal leukotrichia (Figure 1b). The results of the audiological examination identified that the average air conduction hearing threshold of the mother's right ear at 500, 1,000, 2,000, and 3,000 Hz on the audiogram was 50 dB HL (moderate HL), and the hearing threshold of her left ear was 65 dB HL (moderate HL; Figure 2c). The mother of the proband exhibited normal speech. The younger sister of the proband was 10 months old and exhibited a blue right iris, while her left iris was normal (Figure 1c). She passed bilateral optoacoustic emissions and is now undergoing follow‐up auditory examination. The W indexes of the mother and younger sister of the proband were all over 1.95; hence, they have been clinically diagnosed with WS1. The father of the proband had a normal phenotype (Figure 1d). The parents of the proband were informed of the disease condition of the patient, the appropriate expectations for cochlear implantation, and the necessary follow‐up treatment and postoperative rehabilitation training in detail. After ensuring that the parents clearly understood the patient's situation and were prepared for any postoperative treatment, the proband received bilateral cochlear implantation under general anesthesia. The surgery was completed successfully without subsequent complications.

### 3.2 Results of high‐throughput sequencing

**Figure 3:**
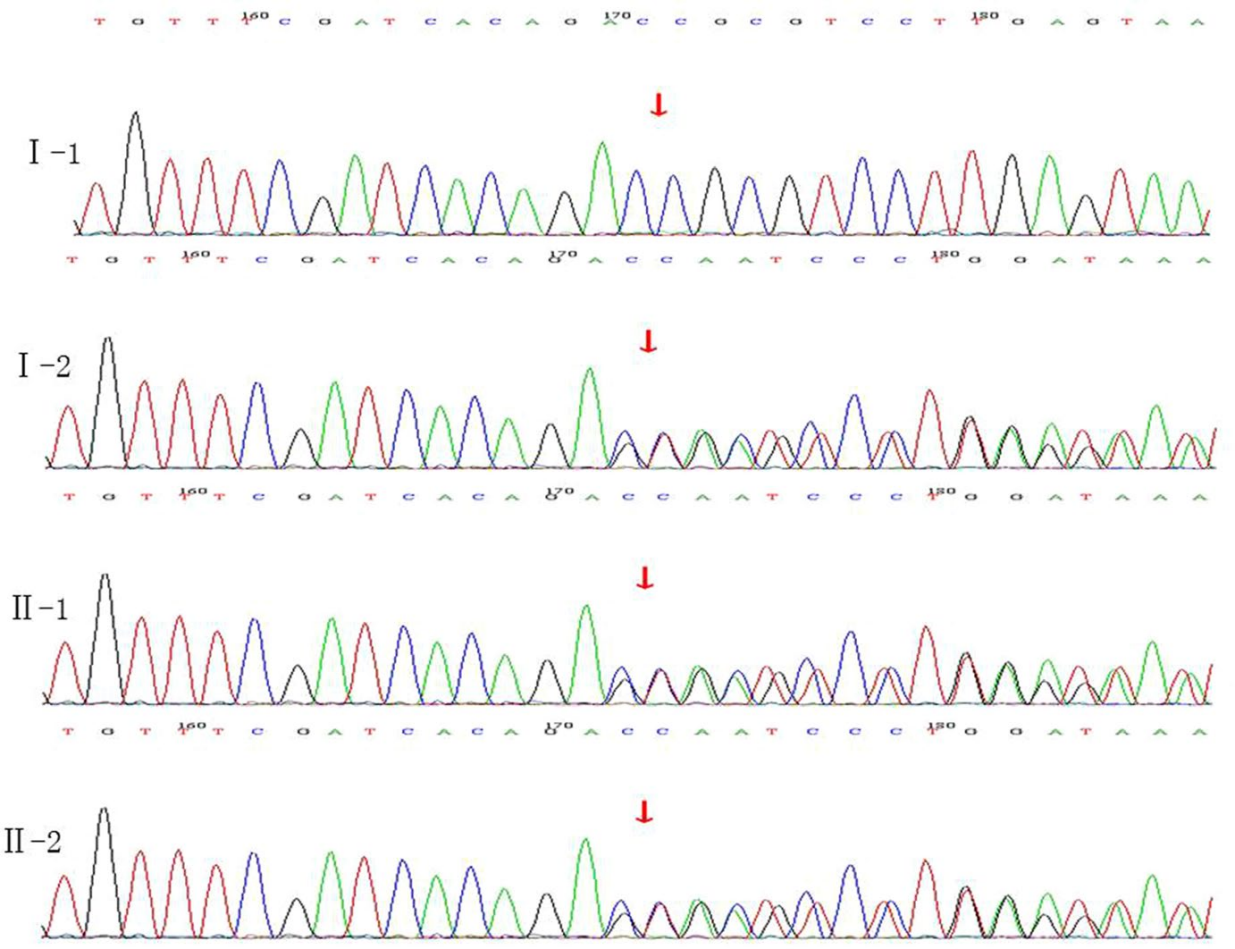
*PAX3*(NM_181457 0.3) sequencing map for the family with WS. The red arrow indicates the site of the base deletion

The *MITF*, *PAX3*, *SOX10*, *SNAI2*, *END3,* and *EDNRB* genes were sequenced with 100% coverage, and the target regions were 9,195 bp long. Sequencing was performed with a 285.5× average sequencing depth and over a 30× average depth. The proportion of loci was 98.15%. The results indicated the heterozygous mutations c.1101A>G (L367L) in *EDNRB*, c.927T>C (H309H) in *SOX10,* and c.420‐424delCGCGGinsTTAC (p.A141Yfs*10) in *PAX3* (Figure3), while no mutations were found in the other three genes by comparison with the 1,000 genomes database. The frequency of the *EDNRB* c.1101A>G mutation was 0.5679 (rs5351), that of the *SOX10* c.927T>C mutation was 0.69 (rs13884), and that of the *PAX3* c.420‐424delCGCGGinsTTAC mutation was 0. No report related to the c.420‐424delCGCGGinsTTAC mutation in *PAX3* was found in the HGMD.

### 3.3 Analysis of the results of Sanger sequencing

**Figure 4:**
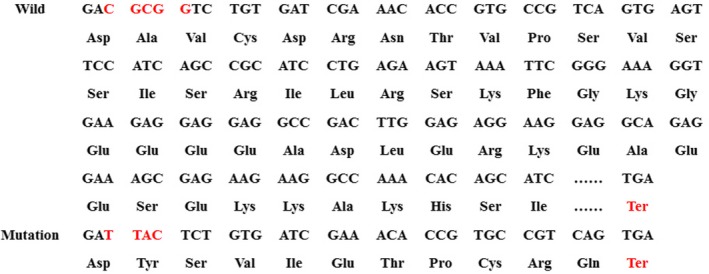
Amino acid coding diagram for the WS family. The red Ter indicates the site of the stop codon. The mutant caused the early termination of the coding sequence

By comparing the sequence of *PAX3* produced from Sanger sequencing to the *PAX3* reference sequence (NM_181457.3), it was found that the proband, his mother and his younger sister all carried the heterozygous mutation c.420‐424delCGCGGinsTTAC in *PAX3*. After analysis, it was revealed that this mutation in the *PAX3* gene was a frameshift mutation that caused changes in the amino acid sequence of the *PAX3* protein from AVCDRNTVPSV to YSVIETPCRQ* (* refers to a stop codon) from amino acids 141–151. The stop codon induced by this mutation resulted in the truncation of the *PAX3* protein that therefore affected protein function (Figure 4). The protein products of the *PAX3* mutation were predicted to be toxic by Mutation Taster software. The father of the proband did not carry the *PAX3* mutation, and no other mutations were detected in any other exons or flanking regions of *PAX3*.

## 4 DISCUSSION

WS1 was the first form of WS to be diagnosed. The first case of WS1 was reported by Japanese scientists in 1989 and was caused by distal inversion in the long arm of chromosome 2 (Ishikiriyama et al., 1989). Later, the causative mutation site for WS1 was located by Tsukamoto et al. in 1992 through fluorescent in situ hybridization (FISH) at 2q35, and the *PAX3* gene was cloned (Tsukamoto et al., 1992). Tassabehji and Baldwin et al. first detected *PAX3* variants and revealed the etiology of WS1 at the molecular level (Baldwin, Hoth, Amos, da‐Silva, & Milunsky, 1992; Tassabehji et al., 1992). *PAX3* is located on chromosome 2 (2q36.1) with a length of 99,110 bp, and it contains 10 exons that are transcribed into 3,112 bp mRNA. The CDS (coding sequence) is located in the region from 382 bp to 1593 bp of the mRNA, which encodes a 5,298 Da protein comprising 479 amino acids. *PAX3* is a transcription factor that plays an important role in embryonic development. There are three highly conservative structural domains in the *PAX3* protein: a pairing‐box domain, a homodomain and a transcription activating domain, and the first two domains work synergistically to bind to DNA.

The clinical manifestations of WS in patients are highly diverse. The proband in this report exhibited a completely bilateral blue iris, profound congenital bilateral sensorineural deafness, broadened inner canthi, a nasal bridge pit, and a wide nasion. His W index was 2.2834 (over 1.95); no musculoskeletal, neurologic, or intestinal anomalies were detected; hence, the patient was diagnosed with WS1. The deafness exhibited by WS patients can be unilateral or bilateral congenital sensorineural deafness or gradual hearing loss, and different auditory phenotypes are highly variable in terms of the level of hearing loss. As mentioned above, even the three individuals carrying the same mutation in the family in this case manifested various phenotypes subject II‐1 exhibited profound deafness, and subject I‐2 exhibited severe dysaudia, while subject II‐2 showed normal auditory ability.

Because of the diversity in the clinical phenotypes and the high genetic heterogeneity of the genes involved, WS is difficult to clinically diagnose. *PAX3* is the main causative gene of WS1, and 90% of WS1 patients carry *PAX3* variants. Conventional methods such as PCR and Sanger sequencing are low‐throughput and unable to examine all suspicious genes in patients; therefore, we initially captured and performed high‐throughput sequencing on target regions of the *PAX3, MITF, SNAI2, EDN3, EDNRB,* and *SOX10* genes and then verified the results through Sanger sequencing. In this report, we performed a single nucleotide polymorphism analysis by comparing peripheral blood samples with the NM_181457.3 reference and confirming that the *PAX3* mutation is located on exon 3 on chromosome 2q36,delCGCGGinsTTAC noted at c.420–424. The mutation in the *PAX3* gene was a frameshift mutation, resulting in protein the triplet codon reading frame is altered and a shifting error in a series of gene coding sequences, changed the amino acid sequence from AVCDRNTVPSV to YSVIETPCRQ* (* refers to the stop codon) from amino acids 141–151. The stop codon was induced N‐terminus to amino acid 151, leading to a large reduction in the *PAX3* protein from 479 amino acids to 150 amino acids. According to the ACMG guidelines, the frameshift mutation was a null variants, which may result in loss of gene function. The frequency of this mutation in the normal population database was “‐”, which means the mutation had a low frequency. It was initially determined as a likely pathogenic mutation. The biological function of mutant protein predicted by software SIFT, PloyPhen_2, and REVEL indicated unknown, unknown, and unknown respectively. Combined with the patient's clinical manifestations, we concluded that the c.420‐424de1CGCGGinsTTAC mutation in *PAX3* is the major molecular pathogenesis of WS1. No report of this mutation was found in the HGMD. The mutations detected in the proband and his sister were came from their mother and were inherited autosomal dominantly. Here, we consider it to be a novel mutation. Until June 2016, 128 *PAX3* variants have been reported in the HGMD. Ninety per cent of them were point mutations and indels, of which missense mutations comprised 50%. A total of 95% of the mutation sites were located in exons 2–6 of *PAX3*, and exon 2 showed the highest mutation frequency. *PAX3* deficiency results in chromocytogenesis, leading to the deficiency of vascularis ductus cochlearis cells, changes in cochlear homeostasis and sensorineural deafness (Kim et al., 2014). Tassabehji et al. (1995) reported that the c.358delG mutation in *PAX3* was a missense and frameshift mutation that changed the amino acid at position 120 from glutamic acid to asparagine and led to a frameshift of 32 amino acids (p.Glu120Asnfs*32). In this report, the proband was received in the hospital due to deafness, and genetic diagnosis was performed with the permission of his parents. The patient manifested typical WS1 phenotypes such as congenital deafness, heterochromic iris, and inner canthus ectopia. Although not all WS patients are deaf, hearing loss is the main phenotype of WS. Sixty per cent of WS1 patients and 90% of WS2 patients exhibit deafness, which can severely negatively affect the patients’ lives and is often an important symptom of concern for parents and doctors. Thus far, there is no effective drug intervention therapy for deafness. Because pigmentary disorders caused by WS have no obvious side effects on the quality of patients’ lives, treatment for deafness is the most concerning question for WS patients. Cochlear implantation is the most effective therapy so far. Since Roland et al. (1998) reported the first case of cochlear implantation in 1998, successive cochlear implantation in WS cases has been reported. The assessment of postoperative effects indicated that cochlear implantation in WS patients was as effective as that in other sensorineural deaf patients, which confirmed that cochlear implantation is a feasible therapy for WS. Additionally, no significant variations in the curative effect of cochlear implantation were observed between WS1 and WS2 patients (Kontorinis, Lenarz, Giourgas, Durisin, & Lesinski‐Schiedat, 2011; de Sousa Andrade et al., 2012). The proband in this report received cochlear implantation in our hospital under general anesthesia. The wound healed well postoperatively, and no complications such as infection or hematoma were observed. The patient was discharged from the hospital 6 days post‐operation, and follow‐up examinations indicated that the patient responded well to voices one month post‐operation. The patient received consistent speech rehabilitation training after leaving the hospital and can now have simple conversations with his parents. A follow‐up examination on 20 November 2018 showed a score of 29 (approximately equal to a 9‐month hearing age) on the MAIS (meaningful auditory integration scale) and a score of 33 (approximately equal to an 18‐month hearing age) on the MUSS (meaningful use of speech scale).

We utilized high‐throughput sequencing to examine all exons and flanking regions of the *PAX3, MITF, SNAI2, EDN3, EDNRB,* and *SOX10* genes in the proband and detected a novel mutation, c.420‐424de1CGCGGinsTTAC, in *PAX3*. Discovering and reporting novel WS causative mutations facilitates the analysis of correlations between WS genotypes and phenotypes, gradually improving the WS‐related genetic variation database for the Chinese population and providing evidence for further genetic consultation and the diagnosis and prenatal diagnosis of WS. However, more studies need to be performed to confirm the relationship between the pathologic genotypes and phenotypes of WS.

## CONFLICT OF INTERESTS

The authors declare that they have no competing interests.
